# Supplementary figures and images for: A whole-genome sequenced control population in northern Sweden reveals subregional genetic differences
Source: PLoS One. 2020 Sep 11;15(9):e0237721. doi: 10.1371/journal.pone.0237721 (PMC7485808; doi:10.1371/journal.pone.0237721)

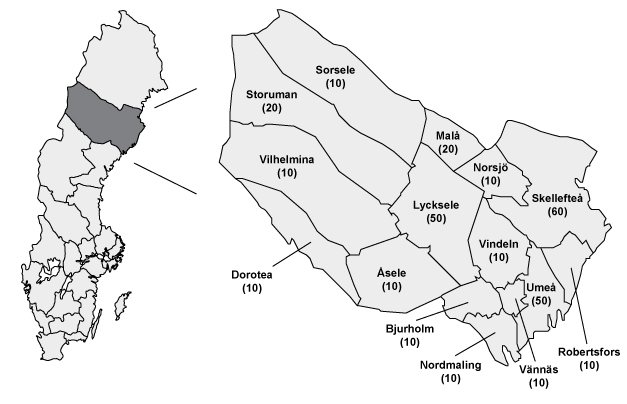

Supplement: S1 Fig — The location of Västerbotten County in Sweden (grey) (left panel). A map of Västerbotten County with the location of all 15 municipalities and their names (right panel). The number of samples drawn from each municipality is indicated within parentheses below the name of the corresponding municipality. (JPG) [file pone.0237721.s001.jpg]

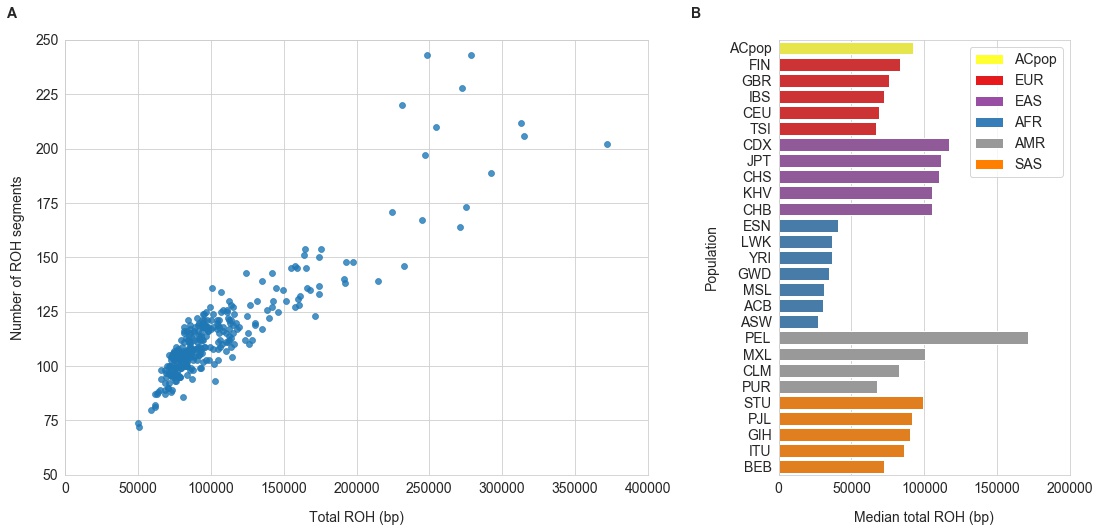

Supplement: S2 Fig — The ROH analysis is performed on a per sample-basis. We plot the total length of ROH segments (in kb) against the number of ROH segments for the ACpop samples (A). In (B), the median total ROH lengths for ACpop and the subpopulations of the 1000g project are visualized. The 1000g project subpopulations in (B) are colored according to superpopulation. (JPG) [file pone.0237721.s002.jpg]

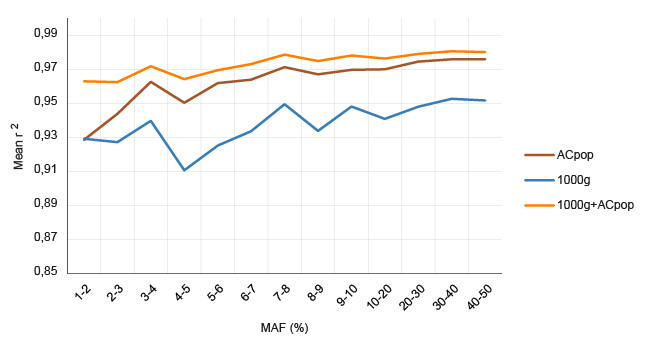

Supplement: S3 Fig — The mean r2 value for imputed and genotyped variants when using 1) only ACpop, 2) only 1000g, and 3) both ACpop and 1000g as the reference panel. The results are binned according to the expected minor allele frequency (MAF). (JPG) [file pone.0237721.s003.jpg]
